# TREAT: systematic and inclusive selection process of genes for genomic newborn screening as part of the Screen4Care project

**DOI:** 10.1186/s13023-025-03692-6

**Published:** 2025-05-15

**Authors:** Christina Saier, Stefaan Sansen, Joanne Berghout, Kathrin Freyler, Moshe Einhorn, Yaron Einhorn, Leslie Matalonga, Sergi Beltran, Antonio Novelli, Rita Selvatici, Fernanda Fortunato, Silvia Montanari, Maria Martinez-Fresno, Gulcin Gumus, Emanuele Agolini, Nicolas Garnier, Alessandra Ferlini, Enrico Bertini, Janbernd Kirschner

**Affiliations:** 1 Department of Neuropediatric and Muscle Disorders, Medical Center, Faculty of Medicine, University of Freiburg, Breisacher Str. 62, 79106 Freiburg, Germany; 2 Department of Laboratory Medicine, Unit II LM-CC, University Hospital Halle (Saale), Halle (Saale), Germany; 3 Sanofi, Diegem, Belgium; 4 Pfizer Inc, Cambridge, MA United States of America; 5 Genoox, Tel Aviv, Israel; 6 Centro Nacional de Análisis Genómico (CNAG), Barcelona, Spain; 7 Laboratory of Medical Genetics, Translational Cytogenomics Research Unit, Bambino Gesù Children Hospital, IRCCS, Rome, Italy; 8 Consorzio Futuro in Ricerca, Ferrara, Italy; 9 Unit of Medical Genetics, Department of Medical Sciences, University of Ferrara, Ferrara, Italy; 10 llumina, Inc, San Diego, CA USA; 11 EURORDIS Rare Disease Europe, Sant Antoni Maria Claret 167, Barcelona, 08025 Spain; 12 Research Unit of Neuromuscular and Neurodegenerative Disease, Bambino Gesù Children Hospital, IRCCS, Rome, Italy; 13 Servier Affaires Médicales, Suresnes, France

**Keywords:** Treatable diseases, Rare diseases, Newborn screening, Genetic, Paediatric, Diagnosis

## Abstract

**Background:**

Genomic newborn screening (gNBS) offers the potential to detect genetic conditions early, enhancing outcomes through timely treatment. It can serve as an additional tool to identify conditions that are not detectable via metabolic screening. The Screen4Care project seeks to develop a systematic approach for selecting treatable rare diseases (RDs) for inclusion in gNBS through the creation of the TREAT-panel.

**Methods:**

A set of six selection criteria containing treatability, clinical validity, age of onset, disease severity, penetrance, and genetic feasibility was applied to a comprehensive list of gene-disease pairs. Genes meeting a defined threshold score were included in the TREAT-panel. This automated scoring process was complemented by expert review from clinicians and patient representatives to ensure clinical relevance and adherence to current medical guidelines.

**Results:**

The initial gene list, derived from multiple data sources, included 484 gene-disease pairs. After applying the scoring system and two rounds of expert curation, a final list of 245 genes was selected. These genes predominantly represent disorders in metabolic, neurological, and immunological categories, with treatability and early disease onset as key inclusion factors.

**Conclusion:**

The Screen4Care TREAT-panel provides a curated, scientifically robust gene set for gNBS, focusing on treatable RDs with early onset and clinical actionability. The panel will be tested in a European pilot project involving approximately 20,000 newborns, contributing to the growing body of evidence for the implementation of next-generation sequencing (NGS) in newborn screening programs.

**Supplementary Information:**

The online version contains supplementary material available at 10.1186/s13023-025-03692-6.

## Background

Over 7000 rare diseases (RDs) [1, 2, 3] have been described, with approximately 72% having a clear genetic origin and about 70% with paediatric onset [4]. Due to the characteristics of RDs and the lack of knowledge about these diseases [4] patients often face a diagnostic odyssey including numerous diagnostic tests and frequent misdiagnoses. A recent survey published results from a Rare Barometer Survey that indicated an average time to diagnosis of almost 5 years [5]. The growing understanding of the genetic basis of RDs leads to the development of an increasing number of therapies, including gene therapy, enzyme replacement and targeted molecular treatments. As many rare diseases are progressive by nature, early diagnosis and treatment are often associated with better outcome for these interventions [6].

Newborn screening (NBS) programs are public health efforts established to screen infants shortly after birth for conditions that are treatable but not always clinically evident yet. Traditionally, metabolite or protein markers in dried blood spots (DBS) are used to screen for these diseases. Advances in genetic testing technologies now allow for DNA sequencing from DBS to test for genetic diseases [6]. Genomic newborn screening (gNBS) provides the opportunity to screen for many conditions for which diagnostic metabolic markers are not available. A prime example of how new treatments combined with the introduction of gNBS has dramatically changed the course of the disease is spinal muscular atrophy. While a few years ago many patients with spinal muscular atrophy died during infancy or required permanent ventilation, early diagnosis through gNBS now often allows these patients to remain free of ventilatory support and achieve motor milestones like sitting and walking [7]. Consequently, an increasing number of countries have introduced a genetic test for spinal muscular atrophy in their national NBS programs [8]. As new treatments are developed at an increasing pace, it becomes challenging for traditional NBS programs to keep up with the inclusion of these diseases.

“Screen4Care: Shortening the path to rare disease diagnosis by using newborn genetic screening and digital technologies” is an IMI-funded research project that combines two pillars: artificial intelligence-guided symptom recognition algorithms and genetic newborn screening [1]. Concerning gNBS, the aim of the Screen4Care consortium is to identify a list of treatable RDs (TREAT-panel-approach) and actionable RDs (ACT-panel approach) and explore their use in a panel-based newborn screening pilot project with around 20,000 infants [1].

This paper describes the development and application of rationale, criteria, and scoring mechanism for the selection of genes whose mutations cause rare, treatable diseases to be included in the TREAT-panel as gNBS tool. To maintain scientific rigour and allow for future adjustments using the same principles, we aimed to use an automated and systematic approach, followed by manual curation by disease experts and patient representatives.

## Methods

### Establishing selection criteria for the TREAT-panel of Screen4Care

The criteria developed for defining the TREAT-panel of Screen4Care build on the principles proposed by Wilson and Jungner in 1968 for the early detection of diseases [9, 10], which have long been considered the gold standard for the selection of RDs for newborn screening [10].

To translate these principles into the genetic testing framework of the TREAT-panel, we identified six selection criteria a gene-disease pair needed to satisfy for inclusion. These criteria are: (1) treatability, (2) clinical validity, (3) age at disease onset, (4) disease severity and (5) penetrance, and (6) genetic technical feasibility. While “treatability” and “genetic technical feasibility” were mandatory criteria for any gene-disease pair to be considered, we employed an automated scoring system with a maximum of 2 points for each of the other criteria, resulting in a maximum score of 8 per gene. The cut off value was defined as 7, meaning that genes scoring lower than 7 were excluded. This also implies that genes with a score of 0 in any of the criteria were excluded. Gene-disease pairs with 7 or 8 points by computational criteria were brought forward as the starting point for the TREAT-panel list, which was then manually reviewed and discussed by clinical and genetics experts to curate further. For further details on the scoring of all criteria see additional file 1.

### Definition of each selection criterion

#### Treatability

Treatability was defined as existence of a medical treatment or intervention that is both efficacious and accessible to patients. The treatment approach should be supported either by regulatory approval or recommendations at the level of medical guidelines. Priority was given for treatments capable of providing transformative benefit to patients by preventing or significantly reducing the development of severe symptoms or morbidity. Acceptable treatments included any drug explicitly approved for a rare indication, approved cell and gene therapies, guideline-established dietary management or nutrient supplementation, and guideline-established bone marrow transplantation. Experimental treatments were excluded as premature, even if emerging evidence suggested efficacy.

To identify potentially treatable diseases, we used the data sources listed in Table 1 to compile a comprehensive initial list of gene/disease pairs, which were then further analysed by our automated scoring system and evaluated by practising clinicians in the relevant domains.

**Table 1:** Description of the data sources used

		Description	Filters	Content	Reference
RxGenes	Treatability	Curated database of treatable genetic diseases. Accessed at https://www.rx-genes.com/ on Feb 20, 2023	Guideline evidence only	211 gene-disease sets	[11]
GTRx	Treatability	Curated tables from the Genome-to-Treatment newborn screening prioritization	Group A category	248 gene-disease sets	[12]
RUSP	Treatability	Recommended Uniform Screening Panel	Deafness, congenital heart diseases were excluded as they are screened by hearing test and pulsoxymetry.	74 diseases	https://www.hrsa.gov/advisory-committees/heritable-disorders/rusp
NBS-Italy and NBS-Germany	Treatability	Diseases currently included in NBS programs in Italy and/or Germany		68 diseases	https://www.eurordis.org/our-priorities/diagnosis/newborn-screening/
EMA-Orphan Drug list	Treatability	EMA approved drugs with label including infants		49 diseases	https://ec.europa.eu/health/documents/community-register/html/reg_od_act.htm?sort=a
ASQM	Treatability, Age-at-onset, Severity		Table S2-category 1	429 genes	[13]

#### Clinical validity

Clinical validity relates to high confidence that pathogenic variants in the identified gene are truly causal, and there must be sufficient evidence that a pathogenic genotype will accurately and predictively identify patients with the disease [14, 15]. Gene-disease pairs satisfying these two criteria were then annotated with additional information to ensure their appropriateness for newborn screening eligibility. Only pathogenic or likely pathogenic variants will be reported.

The Gene Curation Coalition (GeneCC [15]), is a global, multi-institute effort including ClinGen, OMIM, Orphanet, PanelApp and others, created to achieve consensus about gene-disease relationship validity across the field by using a standardized framework for scoring literature and clinical evidence. Gene-disease validity was scored according to the curation conducted by the GeneCC. The complete database of submissions, consisting of 6006 curations for 4704 putatively medically significant genes was downloaded [16]. Each gene-disease relationship was categorized by one or more submitters as “Definitive”, “Strong”, “Moderate”, “Limited”, “Disputed Evidence”, “Refuted Evidence”, “Animal Model Only”, or “No Known Disease Relationship” [17]. Submitter-specific assertions were combined, and the highest scoring curation was used. Gene-disease pairs needed to score “definitive” or “strong” to be eligible for inclusion in the TREAT-panel.

#### Age of onset

To ensure a gene-disease dyad was appropriate for screening and reporting during the newborn period, we evaluated diseases for typical age at onset using Orphanet categorical age-at-onset data and categorical curations from the age-based semi-quantitative metric (ASQM) published by the North Carolina Newborn Exome Sequencing for Universal Screening study [13].

The typical age interval for disease onset was retrieved from Orphanet [18]. One or more age category for each disease were encoded by Orphanet spanning antenatal through elderly. Diseases typically presenting in early childhood (neonatal, infancy, birth, birth-to-childhood, or childhood) were awarded 2 points as having primarily paediatric onset, diseases with onset in adolescence were scored 1 point, and diseases with onset primarily in adulthood were scored 0 points. For cases where Orphanet described a spectrum of ages spanning neonatal through adult for a given disease, the earliest age at onset was selected for our automated approach and scored. Diseases with no data available in either of these two resources were given an automatic scoring of 1.

Additionally, a sub-criterion was applied manually after scores were assembled: treatment should be indicated within the first two years of life for the inclusion of the disease into the panel.

#### Disease severity

For disease severity, 2 points were given for diseases most likely to cause significant health problems. 1 point was scored for diseases with a spectrum of severity or for diseases where the severity was difficult to predict. Diseases not causing significant health problems were scored with 0 points.

The scoring of ASQM for “disease severity” was used as well. Their category “0; no significant morbidity or impairment of quality of life” was put in the Screen4Care category “not causing significant health problem” and scored with 0. The category “1; chronic morbidity (e.g. organ impairment unlikely to cause death, mild-moderate intellectual disability)” was translated into “spectrum of severity, difficult to predict” and scored with 1, while “2; significant morbidity (e.g. significant organ dysfunction, possible/later-onset death or severe neurological involvement)” and “3; sudden or unavoidable childhood death (e.g. fatal arrhythmia; neurodegeneration)” were categorised as “most likely to cause significant health problem” and scored with 2 for Screen4Care. Gene-disease pairs with no severity score in ASQM were assigned a 1.

#### Penetrance

While clinical validity was evaluated above and used as a strict exclusion criterion, a truly pathogenic genotype in a truly causal gene may not always lead to a penetrant, highly morbid phenotype. With a massive increase in sequence data available in medical system biobanks and ostensibly healthy individuals as well as more thorough characterization of pedigrees via cascade testing, it is apparent that genotype-phenotype relationships can be variable, and certain “genotype-positive” patients for certain genes may experience only mild symptoms [19]. Given that our test cohort consists of pre-symptomatic newborns, we sought to incorporate an assessment of penetrance into our scoring criteria to mitigate the risks associated with reporting positive findings in test subjects who may never manifest the disease.

Given the limited availability of information on penetrance and the absence of a gold standard, we relied on data from the BabySeq list. Their categories “low (< 20% of individuals were symptomatic)”, “moderate (20–80% of individuals were symptomatic)” and “high (≥ 80% of individuals were symptomatic)” were translated into the Screen4Care scoring “0; low penetrance (< 20%)”, “1; intermediate penetrance (20–80%)” and “2; penetrance > 80%”. Diseases with no penetrance information available were automatically assigned a score of 1 to avoid exclusion solely based on the lack of penetrance data.

#### Genetic feasibility

The last criterion was genetic feasibility. Genes with a significant number of pathogenic variants annotated in the database and detectable by the comprehensive NGS approach from Screen4Care were included in the TREAT-panel, whereas diseases with non-mendelian inheritance or genes that were not identifiable by the chosen NGS approach were excluded. To minimize the risk of false positives or findings with uncertain significance in the context of newborn screening, we will only report pathogenic or likely pathogenic variants. Positive results will be communicated to participating families only if there are two pathogenic or likely pathogenic variants in cases of autosomal recessive diseases or one such variant in cases of autosomal dominant inheritance. Through this approach, we prioritize findings with a high likelihood of being associated with disease manifestation, thereby minimizing unnecessary anxiety for families.

### TREAT-panel gene selection – automated scoring and ranking

To evaluate gene-disease pairs according to our scoring, we created a harmonization and automated scoring workflow in RStudio. This approach not only adds rigour to the process but also allows for easy reproduction of the workflow at later stages to generate up-to-date TREAT gene panels. Genes were matched to their current Human Gene Nomenclature Committee (HGNC) gene symbol and HGNC ID using the HGNC package [20], and MONDO identifiers were used to standardize disease nomenclature. The code is available on GitHub upon request. For details concerning access and use of the different sources (RxGenes, GTRx, ASQM, NBS programs, and EMA approved orphan drugs) see additional file 2.

After adding the scoring for each gene/disease, all genes scoring lower than 7 were excluded.

### Expert curation

To address missing data on prevalence, disease onset, clinical validity or disease severity required for the automated scoring and ranking rubric, two rounds of expert curation were conducted. These rounds aimed to ensure that the rubric accurately reflected current clinical knowledge and experience. Additionally, the process included input from individuals living with rare diseases (PLWRD) to provide a more inclusive and comprehensive perspective. During the first cycle, clinical experts directly involved in the TREAT-panel development reviewed the list of gene-disease pairs. In the second cycle, the list was shared with the entire Screen4Care consortium, the Screen4Care patient advisory board (see additional file 3), and the Screen4Care scientific advisory board (see additional file 3). During the consultation period, it was possible to propose the inclusion of additional genes or the exclusion of certain genes. Feedback was collected on standardized forms that referred to the predefined selection criteria described above and included references to support requested changes in scoring. (see additional file 4)

## Results

### Initial gene list based on treatability

**Fig. 1:**
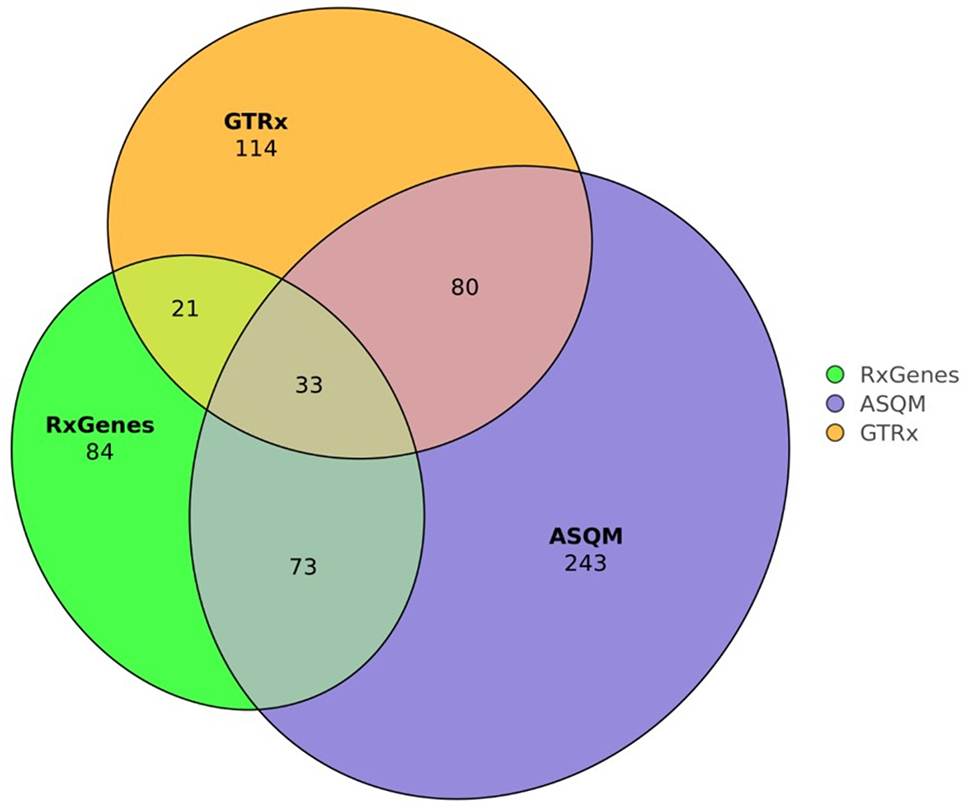
Composition and overlap of the initial TREAT-panel list from various sources. The numbers represent the count of distinct genes within each group

To develop a starting list for treatable diseases we retrieved gene-disease sets from the data sources listed in Table 2. Gene lists and accompanying information from each source were intersected by HGNC ID leading to the selection of 484 unique genes. The overlap between GTRx, RxGenes and ASQM is illustrated in Fig. 1.

### Scoring for clinical validity, age of onset, disease severity and penetrance

As a next step, we applied our scoring for clinical validity, age of onset, disease severity and penetrance. The distribution of the scoring for individual criteria is shown in Table 2. 265 genes were excluded because the total score was “6” or less leaving a final list of 219 genes with a score of “7” or “8”. Scores for clinical validity were high with a score of “2” for 90% of genes, which is most likely due to the sources used for the primary selection of genes. Age of onset was the most determinant criteria, directly excluding 136 diseases based on a score of “0”.

**Table 2:** Distribution of the scoring for the four selection criteria

	0	1	2
Clinical validity	36	11	438
Age of onset	136	0	349
Disease severity	7	311	167
Penetrance	19	190	276

### Expert curation

During the two rounds of expert curation, a total of 34 genes were excluded from the panel by experts from the consortium or members of the patient and scientific advisory boards. T The main reasons for exclusion are detailed in Table 3.

**Table 3:** Reasons for manual exclusion of genes and number of genes excluded for that reason

Reason	Number of genes
No genotype phenotype correlation/not predictable/highly variable	10
Age at onset (not predictable/onset predominantly after two years of age)	17
No evidence that early diagnosis will really change evolution	8
Intermediate/variable penetrance	4
Treatability (majority of phenotypes not treatable/effectiveness of treatment questionable)	8
Treatment availability not in early childhood	2

In addition, 73 gene/disease pairs were proposed for inclusion in the panel by individual experts using a standardized submission form. Following the application of our selection criteria and extensive discussion among clinicians and experts, 60 of these gene/disease pairs were ultimately added to the panel.

**Fig. 2:**
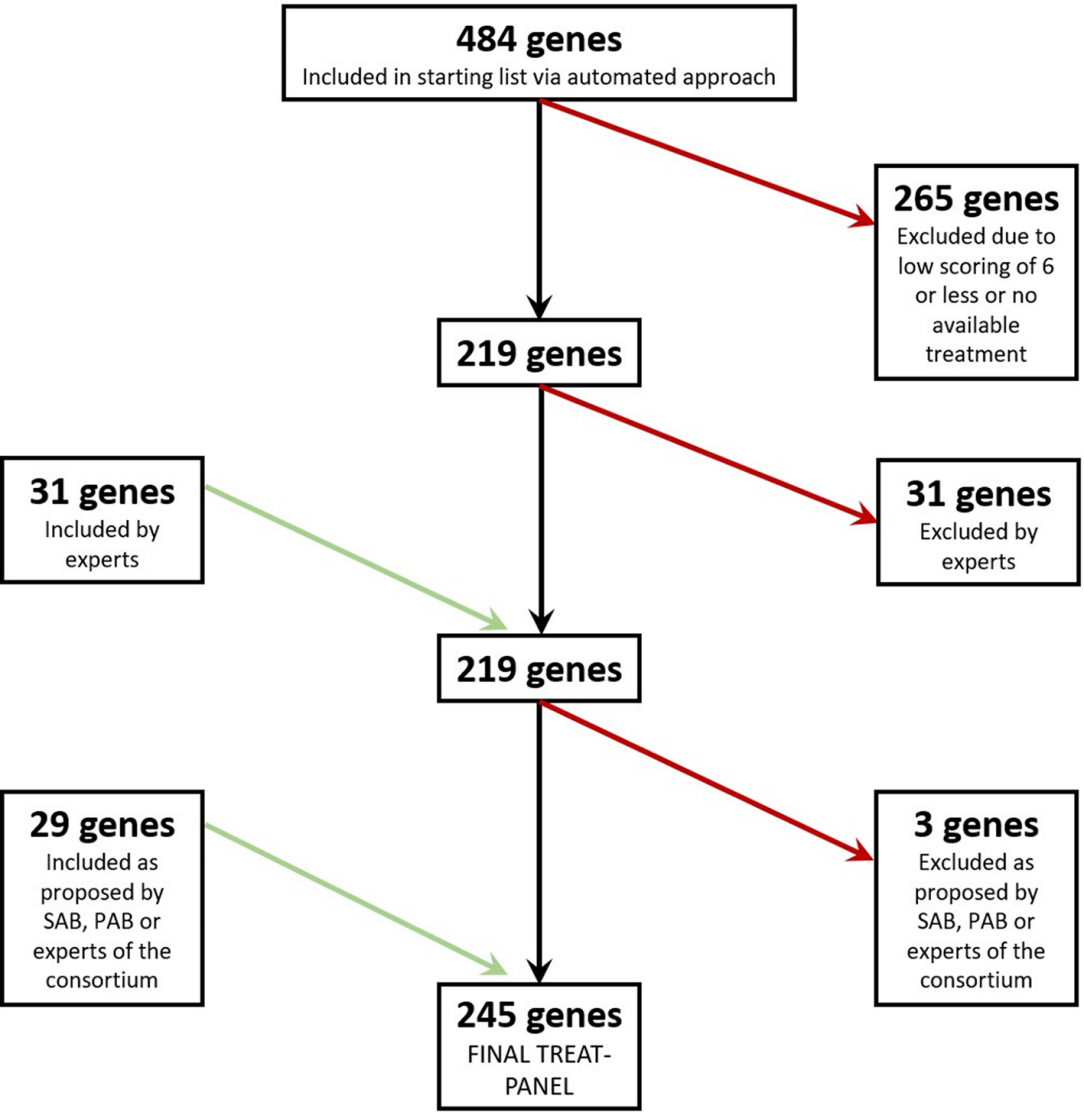
Flowchart for the TREAT-panel selection process. Genes excluded are indicated on the right with red arrows, while genes added during the process are shown on the left with green arrows

An overview on the development of the TREAT-panel, including the added and excluded genes, is presented in Fig. 2.

### Composition of final TREAT-panel

The final TREAT-panel for the Screen4Care project includes 245 different genes (for details, see additional file 4). The largest group of these genes (106) code for metabolic disorders, including mitochondrial, oxidation and lysosomal disorders. Additionally, 33 genes are associated with blood and coagulation disorders, while 29, 26 and 25 genes are related to endocrinological disorders, immunological disorders, and neurological, neurodegenerative, and neuromuscular disorders, respectively. Table 4 summarizes the distribution of genes included in the TREAT-panel according to the different categories of disorders.

**Table 4:** Number of genes included in the TREAT-panel sorted by categories of disorders

Category	Number of genes (total: 245)
Blood and coagulation disorders	33
Cardiological disorders	4
Endocrinological disorders	29
Immunological disorders	26
Kidney disorders	9
Metabolic disorders (including mitochondrial, oxidation, lysosomal disorders, etc.)	106
Neurologic, neurodegenerative and neuromuscular disorders	25
Syndromic disorders	6
Others	7

## Discussion

The development of the TREAT-panel for the Screen4Care project represents a meaningful step forward in the field of genomic newborn screening. Our approach builds on the foundational principles of Wilson and Jungner [10] and adapts them to the modern genetic testing landscape. By systematically integrating treatability, clinical validity, age of onset, disease severity, penetrance, and genetic feasibility into our selection criteria, we have created a robust and scientifically rigorous approach to identifying gene-disease pairs suitable for newborn screening. In addition to gene selection, we will employ standardized operating procedures (SOP) for data analysis to enhance the specificity of our approach. For all genes, we will only report pathogenic or likely pathogenic variants according to the ACMG classification. Due to the challenges associated with the detecting copy number variants (CNVs) with short-read sequencing, we will implement special algorithms and restrict reporting to genes where pathogenic deletions or duplications are well-documented. This comprehensive approach ensures that our screening process is both accurate and clinically relevant. Further, the screening with the TREAT-panel will be piloted in three European countries (Italy, Germany, France), which offers the opportunity to create a common international approach of genomic newborn screenings.

Genomic newborn screening (gNBS) programs have been gaining momentum globally, with various initiatives demonstrating the potential of genomic technologies to expand the scope of traditional newborn screening (NBS). For example, the BabySeq project in the United States has pioneered the use of whole exome sequencing (WES) in newborns, providing valuable insights into the clinical utility of gNBS [21]. The Generation Study in the UK aims to explore the benefits, challenges and practicalities of sequencing and analysing 100,000 newborns for treatable genetic disorders using whole genome sequencing (WGS) [22].

Betzler et al. conducted a comparison of seven published gene-disease lists for gNBS and found substantial variation in total gene count and disease group composition, despite shared selection criteria. They also highlighted that a significant number of genes included in these lists lacked ClinGen curation [23]. More recently, Minten et al. [24]. compared gene lists from 27 different genetic newborn screening programs, revealing that the number of genes analyzed ranged from 134 to 4,299, with only 74 genes included by over 80% of the programs. The Screen4Care gene list was also part of this analysis and demonstrated high concordance with gene lists from other gNBS programs. However, the analysis also identified substantial heterogeneity in the genes included across programs, underscoring the complexity of gene list curation in gNBS initiatives. Currently, there is no single method or data source that can comprehensively guide gene selection, emphasizing the need for further collaborative research to achieve consensus and establish a standardized, evidence-based framework for prioritizing genes and disorders in newborn screening programs.

Compared to other more comprehensive genomic approaches, the Screen4Care TREAT-panel adopts a more targeted and pragmatic approach. For disease selection, it integrates multiple data sources, including existing newborn screening programs and orphan drug repositories, followed by an automated scoring and two rounds of thorough expert review. This approach involves a balance between an in-depth evidence review for each individual gene-disease pair and the broader and less selective approach of WES. While WES offers extensive data that can uncover a wide range of genetic conditions, it also introduces challenges such as the detection of untreatable diseases, the interpretation of incidental findings, variants of uncertain significance (VUS), and the need for extensive resources to analyze, manage and communicate this information.

In contrast, the TREAT-panel is designed to focus on a curated list of treatable RDs, leveraging current knowledge of gene-disease relationships to select only those pairs with strong evidence of treatability and clinical validity. This pragmatic approach allows us to explore the potential of gNBS without being overwhelmed by the complexity associated with broader genomic screening. It aims for a balance between being comprehensive and restrictive, ensuring that the conditions included in the panel are those most likely to benefit from early detection and intervention. Another added value of our targeted sequencing approach is the improved ability to accurately detect CNVs, making their identification more reliable.

Despite the advantages of our automated system, this approach has limitations. One significant challenge is incomplete or missing information in publicly available databases, particularly concerning critical factors such as disease penetrance. For many rare diseases, data on penetrance are sparse or inconsistent, which can lead to uncertainties in scoring and potentially exclude conditions that might otherwise meet our criteria for inclusion.

To address these limitations, expert curation was an essential component of our methodology. Following automated scoring, we conducted two rounds of expert review including our patient and scientific advisory groups. Expert input was particularly crucial in cases where automated scoring was hindered by incomplete data, allowing us to make more informed decisions about which genes to include. This manual review process led to the addition and deletion of a significant number of diseases. Although manual curation was a crucial step in our approach, it is important to acknowledge that conducting an in-depth review of each of the more than 200 gene-disease pairs to the same extent typically employed when evaluating individual diseases for inclusion in national newborn screening programs was not feasible. While the manual curation process was structured, it still carries the risk of overlooking existing evidence for specific gene-disease pairs.

For some genes, there are allelic conditions, and not all of them may meet our selection criteria. Our approach focused on the most common phenotypes, which means that we might also detect allelic conditions, for which early initiation of treatment might not be feasible. Ideally, it would be defined in advance for each individual genetic variant whether it should be reported, but this was not feasible within the Screen4Care project. Moreover, this approach has limitations, as many mutations in rare diseases are novel and not included in public databases.

Additionally, there is a need for ongoing discussion regarding which findings should be reported to parents, carefully balancing the risks of false positives and false negatives. Reporting too many false positives can lead to unnecessary anxiety and additional testing, while missing a true positive could result in delayed diagnosis and treatment, potentially impacting the child’s health. Engaging with a broad range of stakeholders, including healthcare providers, ethicists, and patient advocacy groups, will be crucial in developing guidelines that ensure parents receive clear, accurate, and actionable information from gNBS results. This ongoing dialogue will help establish best practices that prioritize both the health of the newborn and the well-being of the family.

## Conclusion

In conclusion, the Screen4Care TREAT-panel design represents an effort to use the genomic technologies for the early detection and treatment of rare diseases. By combining a systematic methodology with expert curation and community input, we have developed a panel which is both scientifically robust and clinically relevant. As the field of genomics continues to advance, the TREAT-panel pilot project could play a critical role in shortening the diagnostic odyssey for rare disease patients and improving outcomes through earlier and more precise interventions.

## Electronic supplementary material

Below is the link to the electronic supplementary material.

Supplementary Material 1

Supplementary Material 2

Supplementary Material 3

Supplementary Material 4

Supplementary Material 5

## Data Availability

All datasets supporting the content of this manuscript are included within the article and its additional files.

## Abbreviations

ACMG: American college of medical genetics
ASQM: Age-based semi-quantitative metric
CNV: Copy number variants
DBS: Dried blood spots
EMA: European medicines agency
GeneCC: Gene curation coalition
gNBS: Genomic newborn screening
HGNC: Human gene nomenclature committee
NBS: Newborn screening
PLWRD: Persons living with rare diseases
RD: Rare disease
SOP: Standard operating procedure
WES: Whole exome sequencing
WGS: Whole genome sequencing
