# Supplementary material for: TREAT: systematic and inclusive selection process of genes for genomic newborn screening as part of the Screen4Care project
Source: Orphanet J Rare Dis. 2025 May 15;20:231. doi: 10.1186/s13023-025-03692-6 (PMC12082943; doi:10.1186/s13023-025-03692-6)
Supplement: Supplementary file 1 — Supplementary Material 1 [file 13023_2025_3692_MOESM1_ESM.pdf]

Proposal of genes for TREAT-panel

Contact details from person submitting the proposal

Name: Organization:

Email address:

Details on the gene suggested for inclusion on the TREAT-panel

Gene name: Associated diseases:

HGNC symbol for gene: Orphanet-ID of diseases:

Treatment (mandatory)

Please describe available treatment with supporting references (PMID):

Please propose scoring according the TREATpanel selection criteria

Disease onset

X	Score	Definition
○	2	Predominantly paediatric onset of disease
○	1	Spectrum of onset across age groups, difficult to predict onset/limited knowledge about natural history
⊙	0	Mainly adult onset (>18 years)

Disease severity

X	Score	Definition
○	2	Most likely to cause significant health problem
○	1	Spectrum of severity, difficult to predict
⊙	0	Not causing significant health problem

Penetrance

X	Score	Definition
○	2	Penetrance > 80%
○	1	Intermediate penetrance (20-80%)
⊙	0	Low penetrance (< 20%)

Clinical validity

X	Score	Definition
○	2	Known pathogenic variants with clear genotype-phenotype correlation
○	1	Genes with known pathogenic variants and partial genotype/phenotype correlation (as in ultrarare dis)
⊙	0	Genes with only benign or variants of unknown significance, no established geno/phenotype correlation

Additional comments:

This project has received funding from the Innovative Medicines Initiative 2 Joint Undertaking (JU) under grant agreement No. 101034427. The JU receives support from the European Union's Horizon 2020 research and innovation programme and EFPIA. www.imi.europa.eu
