# Supplementary material for: TREAT: systematic and inclusive selection process of genes for genomic newborn screening as part of the Screen4Care project
Source: Orphanet J Rare Dis. 2025 May 15;20:231. doi: 10.1186/s13023-025-03692-6 (PMC12082943; doi:10.1186/s13023-025-03692-6)
Supplement: Supplementary file 2 — Supplementary Material 2 [file 13023_2025_3692_MOESM2_ESM.pdf]

Additional file 1: selection criteria for the TREAT-panel

1. TREATability

Scoring	Definition
YES	Approved drug treatment (EMA) incl. gene therapy and/or other treatment/intervention (drug, diet, bone marrow transplantation, supplements, vitamins, etc) that is recommended by guidelines (at least for a subgroup of the disease)<br>**and**<br>Treatment available in Germany and Italy
NO	All genes/diseases not fulfilling the criteria above<br>☒ Leads to immediate exclusion

2. Disease onset

Scoring	Definition
2	Predominantly paediatric onset of disease
1	Spectrum of onset across age groups, difficult to predict onset/limited knowledge about natural history of disease
0=NO	Mainly adult onset (> 18 years)<br>☒ Leads to immediate exclusion

Subcriterion: Treatment needed within the first two years of life.

3. Disease severity

Scoring	Definition
2	Most likely to cause significant health problem
1	Spectrum of severity, difficult to predict
0	Not causing significant health problem

4. Penetrance

Scoring	Definition
2	Penetrance > 80%
1	Intermediate penetrance (20-80%)
0	Low penetrance (< 20%)

5. Clinical validity

Scoring	Definition
2	known pathogenic variants with clear phenotype-genotype correlation
1	genes with known pathogenic variants and partial genotype/phenotype correlation (as in ultrarare conditions)
0	genes with only benign variants or variants of unknown significance, no established genotype-phenotype correlation

6. Genetic feasibility

Scoring	Definition
YES	significant number of pathogenic variants annotated in database, detectable by our comprehensive NGS approach

NO	Non-mendelian inheritance, not identifiable by chosen NGS Approach<br>☒ Leads to immediate exclusion
