# Supplementary material for: TREAT: systematic and inclusive selection process of genes for genomic newborn screening as part of the Screen4Care project
Source: Orphanet J Rare Dis. 2025 May 15;20:231. doi: 10.1186/s13023-025-03692-6 (PMC12082943; doi:10.1186/s13023-025-03692-6)
Supplement: Supplementary file 3 — Supplementary Material 3 [file 13023_2025_3692_MOESM3_ESM.pdf]

Additional file 2: Access and use of the different sources

RxGenes: under an API license, we accessed the RxGenes database on 20 February 2023 and identified all gene-disease records where "Evidence for Treatment" was equal to "Guideline". All fields for these records were obtained, including the nature of the treatment and a published guideline reference. Data were transformed so that each gene defined a row and all other data were arranged in columns. Genes with more than one database-associated disease were compressed to a single gene-level row, and the gene with the earliest age of onset for the treatable disease was given priority after manually reviewing each pleiotropic gene (n=4 records) for high clinical validity in the named conditions. For example: BRCA in familial breast and ovarian cancer (autosomal dominant) and Fanconi anemia (recessive); HBB in both beta-thalassemia (recessive) and sickle-cell disease (recessive, founder mutation). This yielded 214 distinct genes with evidence for involvement in a treatable genetic disease.

GTRx: Supplementary table 3 was extracted from Kingsmore et al. (2022, PMID: 36007526) "Genotype-to-Treatment" project and data were cleaned and filtered to remove all rows where GTRx curators had categorized a disorder as excluded from newborn sequencing eligibility. Remaining gene-disease pairs had been categorized into "Group A" (n=295 genes), considered suitable based on strong evidence, high likelihood of benefit, and low risk of harm, and "Group B" (n=93 genes) which curators considered to have remaining gaps or uncertainty requiring additional data. Both Group A and Group B genes were combined with the RxGenes table, but only Group A (n=295 genes) was considered TREAT-panel eligible.

ASQM: Supplementary table 2 was extracted from Milko et al 2019 "Age-based Semi-Quantitative Metric" for newborn sequencing and data were cleaned and filtered to monogenic disorders with one row per gene. Work by this group had categorized gene-disease dyads into four categories including (1) pediatric conditions with high actionability, n=409 genes, (2) pediatric conditions with low or no actionability, n=205 genes, (3) adult conditions with high actionability, n=13 genes and (4) adult conditions with low or no actionability, n=16 genes. Category 1 gene-disease pairs were included as TREAT eligible.

Newborn Screening Programs: Conditions currently screened for by biochemical assessment of dried blood spots or other clinical testing were identified from the Recommended Uniform Screening Program (RUSP) website (https://www.hrsa.gov/advisory-committees/heritable-disorders/rusp), a recent

manuscript describing the national program in Italy (https://pubmed.ncbi.nlm.nih.gov/35997437/), and a public health website for Germany (https://www.kinderarzt-berlin-zia.de/en/newborn-screening.html). Disorder names were converted to standardized terminology with MONDO identifiers using the tools available on the Monarch Initiative website (Mungall et al. 2017, PMID: 27899636), and MONDO-annotated "causal genes" for each disease ID were downloaded (data version: February 11, 2023). Multiple newborn screening programs include evaluation for congenital heart disease via pulse oximetry, congenital deafness, and immunodeficiency by T-cell number and function. While these three conditions are extremely clinically important, they are highly heterogeneous with tens to hundreds of genes known to play meaningful roles and influence from non-genetic developmental factors. For TREAT panel gene selection, we excluded these three conditions from this source to focus on more clearly molecularly defined entities.

European Medical Agency (EMA) approved orphan disease drugs: Diseases with an orphan-designated approved medicinal product were identified from EMA website (https://ec.europa.eu/health/documents/community-register/html/reg_od_act.htm?sort=n accessed on November 2, 2022). Search results were curated for genetic diseases (removing rare adult-onset cancers, infectious diseases, neurodegenerative diseases) and limited to diseases for which the label included treatment during the first two years of life. Indications were converted to OMIM disease identifiers, and causal genes were associated through OMIM resources.

Additional file 2: Access and use of the different sources

RxGenes: under an API license, we accessed the RxGenes database on 20 February 2023 and identified all gene-disease records where "Evidence for Treatment" was equal to "Guideline". All fields for these records were obtained, including the nature of the treatment and a published guideline reference. Data were transformed so that each gene defined a row and all other data were arranged in columns. Genes with more than one database-associated disease were compressed to a single gene-level row, and the gene with the earliest age of onset for the treatable disease was given priority after manually reviewing each pleiotropic gene (n=4 records) for high clinical validity in the named conditions. For example: BRCA in familial breast and ovarian cancer (autosomal dominant) and Fanconi anemia (recessive); HBB in both beta-thalassemia (recessive) and sickle-cell disease (recessive, founder mutation). This yielded 214 distinct genes with evidence for involvement in a treatable genetic disease.

GTRx: Supplementary table 3 was extracted from Kingsmore et al. (2022, PMID: 36007526) "Genotype-to-Treatment" project and data were cleaned and filtered to remove all rows where GTRx curators had categorized a disorder as excluded from newborn sequencing eligibility. Remaining gene-disease pairs had been categorized into "Group A" (n=295 genes), considered suitable based on strong evidence, high likelihood of benefit, and low risk of harm, and "Group B" (n=93 genes) which curators considered to have remaining gaps or uncertainty requiring additional data. Both Group A and Group B genes were combined with the RxGenes table, but only Group A (n=295 genes) was considered TREAT-panel eligible.

ASQM: Supplementary table 2 was extracted from Milko et al 2019 "Age-based Semi-Quantitative Metric" for newborn sequencing and data were cleaned and filtered to monogenic disorders with one row per gene. Work by this group had categorized gene-disease dyads into four categories including (1) pediatric conditions with high actionability, n=409 genes, (2) pediatric conditions with low or no actionability, n=205 genes, (3) adult conditions with high actionability, n=13 genes and (4) adult conditions with low or no actionability, n=16 genes. Category 1 gene-disease pairs were included as TREAT eligible.

Newborn Screening Programs: Conditions currently screened for by biochemical assessment of dried blood spots or other clinical testing were identified from the Recommended Uniform Screening Program (RUSP) website (https://www.hrsa.gov/advisory-committees/heritable-disorders/rusp), a recent manuscript describing the national program in Italy (https://pubmed.ncbi.nlm.nih.gov/35997437/), and a public health website for Germany (https://www.kinderarzt-berlin-zia.de/en/newborn-screening.html). Disorder names were converted to standardized terminology with MONDO identifiers using the tools available on the Monarch Initiative website (Mungall et al. 2017, PMID: 27899636), and MONDO-annotated "causal genes" for each disease ID were downloaded (data version: February 11, 2023). Multiple newborn screening programs include evaluation for congenital heart disease via pulse oximetry, congenital deafness, and immunodeficiency by T-cell number and function. While these three conditions are extremely clinically important, they are highly heterogeneous with tens to hundreds of genes known to play meaningful roles and influence from non-genetic developmental factors. For TREAT panel gene selection, we excluded these three conditions from this source to focus on more clearly molecularly defined entities.

European Medical Agency (EMA) approved orphan disease drugs: Diseases with an orphan-designated approved medicinal product were identified from EMA website (https://ec.europa.eu/health/documents/community-register/html/reg_od_act.htm?sort=n accessed on November 2, 2022). Search results were curated for genetic diseases (removing rare adult-onset cancers, infectious diseases, neurodegenerative diseases) and limited to diseases for which the label included treatment during the first two years of life. Indications were converted to OMIM disease identifiers, and causal genes were associated through OMIM resources.
