# Supplementary material for: TREAT: systematic and inclusive selection process of genes for genomic newborn screening as part of the Screen4Care project
Source: Orphanet J Rare Dis. 2025 May 15;20:231. doi: 10.1186/s13023-025-03692-6 (PMC12082943; doi:10.1186/s13023-025-03692-6)
Supplement: Supplementary file 4 — Supplementary Material 4 [file 13023_2025_3692_MOESM4_ESM.pdf]

Additional file 3: Lists of the Scientific Advisory Board and Patient Advisory Board

Scientific Advisory Board

Organisation name	Country	Name	Expertise
International Society of Neonatal Screening	UK	Jim Bonham	President of organization, International Society for Newborn Screening, specialist for metabolic diseases
University of Florence	IT	Giancarlo La Marca	Associate Professor of Clinical Biochemistry
Institute of Child Health UCL	UK	Francesco Muntoni	Professor in Paediatrics Neurology
SERVIER	FR	Nicolas Garnier	Chief Patient Officer
TranslaTUM Technical University of Munich, Institute of Biological and Medical Imaging, Helmholtz Zentrum München	GER	Vasilis Ntziachristos	Chair of Biological Imaging
Genomics England, Queen Mary University of London	UK	Mark Bale	Head of Science Partnerships, consultant for genomics and bioethics
German Foundation for Rare Diseases	GER	Annette Grüters-Kieslich	Professor in Paediatrics with a sub specialty in endocrinology, newborn screening
Smith Family Clinic for Genomic Medicine	UK	David Bick	Medical Director, for the Newborn Genomes Program at Genomics England
Smith Family Clinic for Genomic Medicine	CAN	Hanns Lochmueller	Professor of Neurology, CHEO Research Institute University Ottawa
University of Heidelberg	GER	Franz Schaefer	Pediatric nephrologist

Patient Advisory Board

Organisation name	Name
EURORDIS	Gulcin Gumus
International Gaucher Organisation	Tanya Collin Histed
ALAN - Maladies Rares Luxembourg	Dan Theisen
AFM Telethon/ SMA Europe	Alexandre Mejat
Genetic Alliance UK	Nick Meade
ACHSE	Christine Mundlos
UNIAMO	Simona Bellagambi
Child Rare Disease Support and Research Association Life	Bojana Miroslavljevic
MPS Society	Bob Stevens
World Duchenne Organization	Elizabeth Vroom/Dimitrios Athanasiou
International Pompe Association	Allan Muir
VSOP – Patient advocacy group for rare and genetic disorders	Cor Oosterwijk
