# Supplementary material for: TREAT: systematic and inclusive selection process of genes for genomic newborn screening as part of the Screen4Care project
Source: Orphanet J Rare Dis. 2025 May 15;20:231. doi: 10.1186/s13023-025-03692-6 (PMC12082943; doi:10.1186/s13023-025-03692-6)
Supplement: Supplementary file 5 — Supplementary Material 5 [file 13023_2025_3692_MOESM5_ESM.pdf]

Additional file 4: list of selected treatable rare diseases for the TREAT-panel

Gene-MIM	symbol	ClinGen_valid_disease_title_ Definitive	ClinGen_valid_disease_title_ Strong	TREAT total score
600509	ABCC8	hyperinsulinemic hypoglycemia, familial, 1,familial hyperinsulinism,diabetes mellitus, permanent neonatal 3,monogenic diabetes	transient neonatal diabetes mellitus,diabetes mellitus, noninsulin-dependent,diabetes mellitus, transient neonatal, 2,hypoglycemia, leucine-induced,permanent neonatal diabetes mellitus	8
603214	ABCD4		methylmalonic acidemia with homocystinuria, type cblJ	7
604773	ACAD8	isobutyryl-CoA dehydrogenase deficiency		7
607008	ACADM	medium chain acyl-CoA dehydrogenase deficiency		8
609575	ACADVL	very long chain acyl-CoA dehydrogenase deficiency		8
607809	ACAT1	beta-ketothiolase deficiency		8
102576	ACVR1		fibrodysplasia ossificans progressiva	7
608958	ADA	severe combined immunodeficiency, autosomal recessive, T cell-negative, B cell-negative, NK cell-negative, due to adenosine deaminase deficiency		8
607575	ADA2	CECR1	autoinflammation, vasculitis, immuniodeficiency ,hematological defects syndrome	7
604134	ADAMTS13	congenital thrombotic thrombocytopenic purpura		7
610860	AGL	glycogen storage disease III		7
103320	AGRN	Myasthenic syndrome, congenital, 8, with pre- and postsynaptic defects		8
604285	AGXT	primary hyperoxaluria type 1		8
107323	ALDH7A1	Epilepsy, pyridoxine-dependent		7
612724	ALDOB	hereditary fructose intolerance		8

612866	ALG2	Myasthenic syndrome, congenital, 14, with tubular aggregates		8
607905	ALG14	Myasthenic syndrome, congenital, 15, without tubular aggregates		8
171760	ALPL	hypophosphatasia,infantile hypophosphatasia,childhood hypophosphatasia,adult hypophosphatasia		7
608313	ARG1	hyperargininemia		7
607574	ARSA	metachromatic leukodystrophy,metachromatic leukodystrophy, juvenile form		8
611542	ARSB	mucopolysaccharidosis type 6		8
608310	ASL	argininosuccinic aciduria		8
603470	ASS1	citrullinemia type I		8
605239	ATP6V0A4	renal tubular acidosis, distal, 2, with progressive sensorineural hearing loss		8
192132	ATP6V1B1	renal tubular acidosis, distal, 2, with progressive sensorineural hearing loss		7
300011	ATP7A	X-linked distal spinal muscular atrophy type 3,Menkes disease	occipital horn syndrome	8
606882	ATP7B	Wilson disease		8
600529	AUH	3-methylglutaconic aciduria type 1		7
608348	BCKDHA	maple syrup urine disease type 1A,maple syrup urine disease		8
248611	BCKDHB	maple syrup urine disease type 1B,maple syrup urine disease		8
614901	BCKDK	branched chain keto acid dehydrogenase kinase defect		8
609019	BTD	biotinidase deficiency		7
300300	BTK	Bruton-type agammaglobulinemia		7
611492	CA2	autosomal recessive osteopetrosis 3		7
114205	CACNA1C	Timothy Syndrome, Long QT syndrome		7
613381	CBS	classic homocystinuria		8
300386	CD40LG	hyper-IgM syndrome type 1		8
602421	CFTR	cystic fibrosis		8

118490	CHAT	Myasthenic syndrome, congenital, 6, presynaptic		8
100690	CHRNA1		lethal multiple pterygium syndrome,congenital myasthenic syndrome 1A,myasthenic syndrome, congenital, 1B, fast-channel	7
100710	CHRNB1		congenital myasthenic syndrome 2A,congenital myasthenic syndrome 2C,congenital myasthenic syndrome 1A,postsynaptic congenital myasthenic syndrome	7
100720	CHRND		congenital myasthenic syndrome 3A,congenital myasthenic syndrome 3C,congenital myasthenic syndrome 3B,postsynaptic congenital myasthenic syndrome	7
100725	CHRNE	congenital myasthenic syndrome	congenital myasthenic syndrome 4A,congenital myasthenic syndrome 4B,congenital myasthenic syndrome 4C,postsynaptic congenital myasthenic syndrome	7
120150	COL1A1	Ehlers-Danlos syndrome, arthrochalasis type,Caffey disease,osteogenesis imperfecta type 2,osteogenesis imperfecta type 1,osteogenesis imperfecta type 3		8
120350	COL1A2	ehlers-danlos syndrome, arthrochalasia type, 2	Ehlers-Danlos syndrome, cardiac valvular type	7
120350	COL13A1	Myasthenic syndrome, congenital, 19		8
603033	COLQ	Myasthenic syndrome, congenital, 5		8
609825	COQ2	coenzyme Q10 deficiency, primary, 1		8
614647	COQ6		familial steroid-resistant nephrotic syndrome with sensorineural deafness	8
615567	COQ8B		nephrotic syndrome, type 9	8
608307	CPS1	carbamoyl phosphate synthetase I deficiency disease		8

600528	CPT1A	carnitine palmitoyl transferase 1A deficiency		8
600650	CPT2	carnitine palmitoyltransferase II deficiency	carnitine palmitoyl transferase II deficiency, severe infantile form,carnitine palmitoyl transferase II deficiency, neonatal form,carnitine palmitoyl transferase II deficiency, myopathic form	8
606272	CTNS	cystinosis,nephropathic cystinosis	juvenile nephropathic cystinosis,ocular cystinosis	8
118485	CYP11A1		Congenital adrenal insuffiency with 46, XY sex reversal OR 46,XY disorder of sex development-adrenal insufficiency due to CYP11A1 deficiency	7
610613	CYP11B1	congenital adrenal hyperplasia due to 11-beta-hydroxylase deficiency	glucocorticoid-remediable aldosteronism	8
613815	CYP21A2	classic congenital adrenal hyperplasia due to 21-hydroxylase deficiency		8
606530	CYP27A1	cerebrotendinous xanthomatosis		7
609506	CYP27B1		vitamin D-dependent rickets, type 1A	7
248610	DBT	maple syrup urine disease type 2,maple syrup urine disease		8
605988	DCLRE1C	severe combined immunodeficiency due to DCLRE1C deficiency		8
107930	DDC	aromatic L-amino acid decarboxylase deficiency		7
300377	DMD	Duchenne and Becker muscular dystrophy,Becker muscular dystrophy,dilated cardiomyopathy 3B,Duchenne muscular dystrophy,progressive muscular dystrophy		8
611432	DOCK8	combined immunodeficiency due to DOCK8 deficiency		7
610285	DOK7	Myasthenic syndrome, congenital, 10		8
191350	DPAGT1	congenital myasthenic syndrome 13,hematopoietic stem cell kinetics, control of	DPAGT1-congenital disorder of glycosylation	7

606759	DUOX2	thyroid dyshormonogenesis 6		8
612772	DUOXA2		congenital hypothyroidism	8
130130	ELANE	neutropenia		7
608053	ETFA	multiple acyl-CoA dehydrogenase deficiency		8
130410	ETFB	multiple acyl-CoA dehydrogenase deficiency		8
231675	ETFDH	multiple acyl-CoA dehydrogenase deficiency		8
134570	F13A1	FXIII A subunit deficiency		8
176930	F2	thrombophilia due to thrombin defect,congenital prothrombin deficiency		8
613878	F7	FVII deficiency - bleeding disorder		8
300841	F8	hemophilia A		8
300746	F9	hemophilia B		8
613871	FAH	tyrosinemia type I		8
607139	FANCA	Fanconi anemia complementation group A	Fanconi anemia	8
300515	FANCB	Fanconi anemia complementation group B	VACTERL association, X-linked, with or without hydrocephalus	8
613899	FANCC	Fanconi anemia complementation group C	Fanconi anemia	8
613984	FANCD2	Fanconi anemia complementation group D2		8
613976	FANCE	Fanconi anemia complementation group E		8
613897	FANCF	Fanconi anemia complementation group F		8
602956	FANCG	Fanconi anemia complementation group G		8
611360	FANCI	Fanconi anemia complementation group I		8
608111	FANCL	Fanconi anemia complementation group L		8
607901	FERMT3	leukocyte adhesion deficiency 3		8

134820	FGA	familial dysfibrinogenemia,congenital afibrinogenemia,congenital fibrinogen deficiency	thrombophilia	7
134830	FGB	congenital fibrinogen deficiency	thrombophilia	7
134934	FGFR3	Achondroplasia		8
134850	FGG	congenital fibrinogen deficiency	thrombophilia	7
600838	FOXN1	T-cell immunodeficiency, congenital alopecia, and nail dystrophy		7
300292	FOXP3	immune dysregulation-polyendocrinopathy-enteropathy-X-linked syndrome		7
613742	G6PC1	glycogen storage disease due to glucose-6-phosphatase deficiency type IA		8
305900	G6PD	anemia, nonspherocytic hemolytic, due to G6PD deficiency		7
606800	GAA	glycogen storage disease II		8
606890	GALC	Krabbe disease		8
604313	GALK1	galactokinase deficiency		7
612222	GALNS	mucopolysaccharidosis type 4A		7
606999	GALT	galactosemia,classic galactosemia		8
601240	GAMT	GAMT deficiency		7
305371	GATA1	GATA1-Related X-Linked Cytopenia		7
602360	GATM	AGAT deficiency		7
606463	GBA1	Gaucher disease,Gaucher disease perinatal lethal,Parkinson disease	Gaucher disease type I,Gaucher disease type III,Gaucher disease-ophthalmoplegia-cardiovascular calcification syndrome,Gaucher disease type II,late-onset Parkinson disease	8
608801	GCDH	glutaryl-CoA dehydrogenase deficiency		8
600225	GCH1	Dystonia, DOPA-responsive		7

138079	GCK	maturity-onset diabetes of the young type 2,monogenic diabetes	diabetes mellitus, noninsulin-dependent,hyperinsulinism due to glucokinase deficiency,permanent neonatal diabetes mellitus 1,transient neonatal diabetes mellitus	8
138292	GFPT1	Myasthenia, congenital, 12, with tubular aggregates		8
138130	GLUD1	hyperinsulinism-hyperammonemia syndrome		7
615320	GMPPB	Congenital myasthenic syndrome due to a defect of glycosylation caused by pathogenic variants in GMPPB		8
139320	GNAS	pseudohypoparathyroidism type 1B,ACTH-independent macronodular adrenal hyperplasia 1,pseudohypoparathyroidism type 1A,McCune-Albright syndrome	pseudohypoparathyroidism type 1C	7
611499	GUSB	mucopolysaccharidosis type 7		8
601609	HADH	3-hydroxyacyl-CoA dehydrogenase deficiency,hyperinsulinism due to short chain 3-hydroxylacyl-CoA dehydrogenase deficiency	hyperinsulinemic hypoglycemia, familial, 4	8
600890	HADHA	long chain 3-hydroxyacyl-CoA dehydrogenase deficiency	mitochondrial trifunctional protein deficiency	8
143450	HADHB	mitochondrial trifunctional protein deficiency		8
141800	HBA1	alpha thalassemia	erythrocytosis, familial, 7	7
141850	HBA2		erythrocytosis, familial, 7	7
141900	HBB	sickle cell anemia (before named as: beta thalassemia,beta-thalassemia HBB/LCRB)	erythrocytosis, familial, 6,Heinz body anemia	8
609018	HLCS	holocarboxylase synthetase deficiency		8
613898	HMGCL	3-hydroxy-3-methylglutaric aciduria		8
609695	HPD	tyrosinemia type III	hawkinsinuria	7
300823	IDS	mucopolysaccharidosis type 2		7
252800	IDUA	mucopolysaccharidosis type 1,Scheie syndrome	Hurler syndrome,Hurler-Scheie syndrome	8

147440	IGF1	growth delay due to insulin-like growth factor type 1 deficiency		7
300137	IGSF1	X-linked central congenital hypothyroidism with late-onset testicular enlargement		7
308380	IL2RG	T-B+ severe combined immunodeficiency due to gamma chain deficiency		8
146661	IL7R	immunodeficiency 104		7
176730	INS	isolated permanent neonatal diabetes mellitus 4 (PNDM)		7
600065	ITGB2	leukocyte adhesion deficiency 1		8
607036	IVD	isovaleric acidemia		8
600173	JAK3	T-B+ severe combined immunodeficiency due to JAK3 deficiency		8
152427	KCNH2	long QT syndrome 2,long QT syndrome,short QT syndrome		7
600937	KCNJ11	hyperinsulinemic hypoglycemia, familial, 2,diabetes mellitus, transient neonatal, 3,monogenic diabetes	diabetes mellitus, permanent neonatal 2,diabetes mellitus, noninsulin-dependent,maturity-onset diabetes of the young type 13,hyperinsulinemic hypoglycemia	8
607542	KCNQ1	Jervell and Lange-Nielsen syndrome 1,Jervell and Lange-Nielsen syndrome,long QT syndrome	short QT syndrome	8
600577	LHX3	non-acquired combined pituitary hormone deficiency with spine abnormalities		8
613497	LIPA	lysosomal acid lipase deficiency		8
612625	LMBRD1	methylmalonic aciduria and homocystinuria type cblF		7
604270	LRP4	Cenani-Lenz syndactyly syndrome	congenital myasthenic syndrome 17	7
606897	LYST	Chediak-Higashi syndrome		8
609458	MAN2B1	alpha-mannosidosis		7
606761	MLYCD	malonic aciduria		8
607481	MMAA	methylmalonic aciduria, cblA type		8

607568	MMAB	methylmalonic aciduria, cblB type		8
609831	MMACHC	methylmalonic aciduria and homocystinuria type cblC		8
611935	MMADHC	methylmalonic aciduria and homocystinuria type cblD,inborn disorder of cobalamin metabolism and transport		8
609058	MMUT	methylmalonic aciduria due to methylmalonyl-CoA mutase deficiency		8
154550	MPI	MPI-congenital disorder of glycosylation,SRD5A3-congenital disorder of glycosylation		7
607093	MTHFR	homocystinuria due to methylene tetrahydrofolate reductase deficiency		7
156570	MTR	methylcobalamin deficiency type cblG		8
602568	MTRR	methylcobalamin deficiency type cblE		8
601296	MUSK	Myasthenic syndrome, congenital, 9, associated with acetylcholine receptor deficiency		8
251170	MVK		porokeratosis of Mibelli	7
608300	NAGS	hyperammonemia due to N-acetylglutamate synthase deficiency		8
602667	NBN	Nijmegen breakage syndrome		8
611290	NHEJ1	Cernunnos-XLF deficiency		8
600635	NKX2-1	hereditary progressive chorea without dementia,brain-lung-thyroid syndrome		7
300461	OTC	ornithine carbamoyltransferase deficiency		8
612349	PAH	phenylketonuria		8
232000	PCCA	propionic acidemia		8
232050	PCCB	propionic acidemia		8
610564	PDSS2	coenzyme Q10 deficiency, primary, 3		8
300550	PHEX	X-linked Hypophosphataemia		8
300798	PHKA2	glycogen storage disease IXa1		8

172490	PHKB		glycogen storage disease IXb	7
172471	PHKG2		glycogen storage disease IXc	7
602839	PIK3CD	immunodeficiency 14b, autosomal recessive,immunodeficiency 14	activated PI3K-delta syndrome	7
171833	PIK3R1	SHORT syndrome,agammaglobulinemia 7, autosomal recessive,immunodeficiency 36	agammaglobulinemia	7
609712	PKLR	pyruvate kinase deficiency of red cells		7
604436	PLPBP	vitamin B6 responsive epilepsy		8
601785	PMM2	congenital disorder of glycosylation type I,SRD5A3-congenital disorder of glycosylation	PMM2-congenital disorder of glycosylation	7
603287	PNPO	pyridoxal phosphate-responsive seizures		8
173110	POU1F1	multiple pituitary hormone deficiency		7
170280	PRF1	Hemophagocytic lymphohistiocytosis, familial, 2		7
601538	PROP1	pituitary hormone deficiency, combined, 2		8
610936	PSAT1	neurometabolic disorder due to serine deficiency	Neu-Laxova syndrome 1	7
612719	PTS	BH4-deficient hyperphenylalaninemia A		8
612676	QDPR	dihydropteridine reductase deficiency		8
603868	RAB27A	Griscelli syndrome type 2		7
179615	RAG1	Omenn syndrome,recombinase activating gene 1 deficiency	severe combined immunodeficiency, autosomal recessive, T cell-negative, B cell-negative, NK cell-positive	8
179616	RAG2	recombinase activating gene 2 deficiency	Omenn syndrome	8
601592	RAPSN	fetal akinesia deformation sequence 1	congenital myasthenic syndrome 11	7
614041	RB1	hereditary retinoblastoma,retinoblastoma		7

164761	RET	multiple endocrine neoplasia type 2B,multiple endocrine neoplasia type 2A		8
180069	RPE65	Leber congenital amaurosis 9,RPE65-related recessive retinopathy	RPE65-related dominant retinopathy	7
604175	RPL11		Diamond-Blackfan anemia 7	7
180468	RPL35A		Diamond-Blackfan anemia 5	7
603634	RPL5		Diamond-Blackfan anemia 6	7
603632	RPS10	Diamond-Blackfan anemia	Diamond-Blackfan anemia 9	7
180472	RPS17		Diamond-Blackfan anemia 4	7
603474	RPS19	Diamond-Blackfan anemia 1		7
602412	RPS24	Diamond-Blackfan anemia 3,Diamond-Blackfan anemia		7
603701	RPS26	Diamond-Blackfan anemia 10		7
603658	RPS7		Diamond-Blackfan anemia 8	7
607444	SBDS	Shwachman-Diamond syndrome 1,Shwachman-Diamond syndrome		7
603967	SCN4A	hypokalemic periodic paralysis, type 1,hyperkalemic periodic paralysis,paramyotonia congenita of Von Eulenburg,SCN4A-related myopathy, autosomal recessive	congenital myasthenic syndrome 16,hypokalemic periodic paralysis, type 2,congenital myopathy	7
600163	SCN5A	long QT syndrome 3,Brugada syndrome 1,Brugada syndrome, dilated cardiomyopathy, familial long QT syndrome	progressive familial heart block, type 1A	7
600228	SCNN1A	autosomal recessive pseudohypoaldosteronism type 1	bronchiectasis with or without elevated sweat chloride 2,pseudohypoaldosteronism type 1	8
600760	SCNN1B		bronchiectasis with or without elevated sweat chloride 1,pseudohypoaldosteronism type 1,Liddle syndrome 1	8

600761	SCNN1G	Liddle syndrome	bronchiectasis with or without elevated sweat chloride 3,Liddle syndrome 2,pseudohypoaldosteronism type 1	8
300490	SH2D1A		X-linked lymphoproliferative syndrome	7
600968	SLC12A3		Gitelman syndrome	7
600682	SLC16A1		ketoacidosis due to monocarboxylate transporter-1 deficiency	7
600336	SLC18A3	Myasthenic syndrome, congenital, 21, presynaptic		8
606152	SLC19A3	biotin-responsive basal ganglia disease,Leigh syndrome		8
603377	SLC22A5	systemic primary carnitine deficiency disease		7
190315	SLC25A1	Myasthenic syndrome, congenital, 23, presynaptic		8
603859	SLC25A13	citrin deficiency		8
603861	SLC25A15	ornithine translocase deficiency		7
613698	SLC25A20	carnitine-acylcarnitine translocase deficiency		8
126650	SLC26A3	congenital secretory chloride diarrhea 1		7
605646	SLC26A4	autosomal recessive nonsyndromic hearing loss 4,Pendred syndrome		7
138140	SLC2A1	encephalopathy due to GLUT1 deficiency,childhood onset GLUT1 deficiency syndrome 2,GLUT1 deficiency syndrome	dystonia 9	8
602671	SLC37A4	glycogen storage disease type 1 due to SLC37A4 mutation	glycogen storage disease Ib	7
611672	SLC46A1	hereditary folate malabsorption		8
607882	SLC5A5	familial thyroid dyshormonogenesis 1		8
613350	SLC5A7	Myasthenic syndrome, congenital, 20, presynaptic		8
607882	SLC52A2	Brown-Vialetto-Van Laere syndrome 2		8
608761	SLC52A3	Brown-Vialetto-Van Laere syndrome 1		8

300036	SLC6A8	creatine transporter deficiency		7
603593	SLC7A7	lysinuric protein intolerance		7
600354	SMN1	spinal muscular atrophy,spinal muscular atrophy, type 1	spinal muscular atrophy, type II,spinal muscular atrophy, type III,spinal muscular atrophy, type IV	7
607608	SMPD1	c("Niemann-Pick disease", "Niemann-Pick disease type A", "acid sphingomyelinase deficiency")	Niemann-Pick disease type B	8
182125	SPR	dopa-responsive dystonia due to sepiapterin reductase deficiency		7
600617	STAR	congenital lipoid adrenal hyperplasia due to STAR deficency		8
605014	STX11		familial hemophagocytic lymphohistiocytosis 4	7
601717	STXBP2	familial hemophagocytic lymphohistiocytosis 5		7
600104	SYT2	Myasthenic syndrome, congenital, 7A & 7B , presynaptic		8
613018	TAT	tyrosinemia type II		7
604592	TCIRG1	autosomal recessive osteopetrosis	autosomal recessive osteopetrosis 1	7
188450	TG		thyroid dyshormonogenesis 3	8
191290	TH	tyrosine hydroxylase deficiency,TH-deficient dopa-responsive dystonia		8
190120	THRA	congenital nongoitrous hypothyroidism 6		7
188250	TK2		Mitochondrial DNA depletion syndrome 2	8
612418	TMEM70	mitochondrial complex V (ATP synthase) deficiency nuclear type 2,mitochondrial disease		7
606765	TPO		thyroid dyshormonogenesis 2A	8
607998	TPP1	neuronal ceroid lipofuscinosis 2,neuronal ceroid lipofuscinosis		8
188540	TSHB	isolated thyroid-stimulating hormone deficiency		8

603372	TSHR	hypothyroidism due to TSH receptor mutations,familial gestational hyperthyroidism		8
608897	UNC13D	familial hemophagocytic lymphohistiocytosis 3		8
185880	VAMP1	Myasthenic syndrome, congenital, 25		8
613160	VWF	Type 3 VWD - lack of VWF		8
300392	WAS	Wiskott-Aldrich syndrome,X-linked severe congenital neutropenia		7
300079	XIAP	Lymphoproliferative syndrome, X-linked, 2		7
176947	ZAP70	combined immunodeficiency due to ZAP70 deficiency		8
